# Folliculo-stellate cells of "true dendritic" type are involved in the inflammatory microenvironment of tumor immunosurveillance of pituitary adenomas

**DOI:** 10.1186/1746-1596-2-20

**Published:** 2007-06-27

**Authors:** Istvan Vajtai, Andreas Kappeler, Rahel Sahli

**Affiliations:** 1 Section of Neuropathology, Institute of Pathology, University of Bern, Switzerland; 2 Laboratory of Diagnostic Immunohistochemistry, Institute of Pathology, University of Bern, Switzerland; 3 Department of Endocrinology and Diabetology, University Hospital (Inselspital) Bern, Switzerland

## Abstract

Folliculo-stellate cells are a nonendocrine, sustentacular-like complementary population of the anterior pituitary. They currently are considered as functionally and phenotypically heterogeneous, with one subpopulation of folliculo-stellate cells possibly representing resident adenohypophyseal macrophages. We took advantage of a limited T-cell mediated inflammatory reaction selectively involving tumor tissue in three cases of pituitary adenoma (2 prolactin cell adenomas, and 1 null cell adenoma) to test the hypothesis whether some folliculo-stellate cells within inflammatory foci would also assume monocytic/dendritic properties. Immunohistochemical double labeling for S-100 protein and the class II major histocompatibility antigen HLA-DR indeed showed several arborized cells to coexpress both epitopes. These were distributed both amidst adenomatous acini and along intratumoral vessels, and were morphologically undistinguishable from conventional folliculo-stellate cells. On the other hand, markers of follicular dendritic cells (CD21) and Langerhans' cells (CD1a) tested negative. Furthermore, no S-100/HLA-DR coexpressing folliculo-stellate cells were seen in either peritumoral parenchyma of the cases in point nor in control pituitary adenomas lacking inflammatory reaction. These findings suggest that a subset of folliculo-stellate cells may be induced by an appropriate local inflammatory microenvironment to assume a dendritic cell-like immunophenotype recognizable by their coexpression of S-100 protein and HLA-DR. By analogy with HLA-DR expressing cells in well-established extrapituitary inflammatory constellations, we speculate that folliculo-stellate cells with such immunophenotype may actually perform professional antigen presentation. A distinctly uncommon finding in pituitary adenomas, lymphocytic infiltrates may therefore be read as a manifestation of tumoral immunosurveillance.

## Background

Folliculo-stellate cells (FSCs) are primarily non-hormone secreting accessory elements contributing some 5–10% of cells of the anterior pituitary where they both commingle and functionally interact with the endocrine population proper [1].

Recent evidence has been involving FSCs with facets of adenohypophyseal machinery as manifold as paracrine regulation, cellular turnover, and neuro-immune crosstalk [2-4]. Accordingly, the eponymous arborized morphology of FSCs is becoming but a descriptive umbrella for interstitial cells to be subsumed under, the functions, respective immunophenotypes and indeed histogenesis of which are felt to be nothing less than uniform. Of late, FSCs have been postulated to descend from a common neuro-hematopoietic precursor to eventually diversify along one of three lineages: i.e., "classical/epithelial cell-like"; "classical/astrocyte-like"; and "true dendritic cell-like" [1]. While immunopositivity for S-100 protein is shared by all three derivatives, specific commitment to either epithelial, glial or monocytic/dendritic properties has been shown to entail alternative coexpression of cytokeratin(s), glial fibrillary acidic protein (GFAP), and MHC class II surface antigens, respectively. Several aspects of the glial-epithelial avatars of FSCs have been documented in the context of adenohypophyseal cell cycle – in particular by pioneering observations by Horvath and Kovacs on both nonneoplastic and adenomatous human pituitaries [4,5]. Conversely, data regarding the "true dendritic cell-like" (DC) subset of FSCs, as defined above, are largely restricted to nontumorous human autopsy material and transplantation experiments in mice [6,7].

## Findings

**Figure 1:**
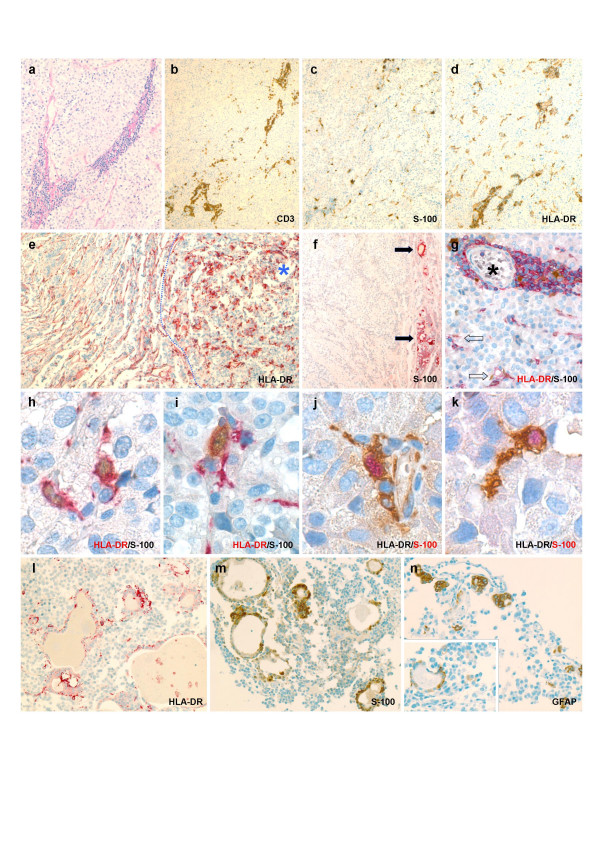
Microphotographs from Case 3 (**a **through **d**) to illustrate basic pattern of inflammatory reaction common to all three adenomas in this report: i.e., angiocentric infiltrates of T lymphocytes (**b**); colonization of tumor interstitium by arborized cells immunoreactive for S-100 protein (**c**) and HLA-DR (**d**), the respective staining patterns of which are felt to be largely overlapping. As exemplified by index Case 1 (**e**), dendritic cell reaction selectively involves the adenoma compartment (*), while only native fibrohistiocytic elements of peritumoral pituitary stain for HLA-DR. Lesional interface is highlighted by dotted line. Conventional gonadotroph cell adenoma from the control series (**f**) is devoid of S-100^+ ^dendritic cells. Arrows point to activated folliculo-stellate cells in adjacent nontumorous acini to technically validate staining reaction. Overview of double immunostained inflammatory focus (**g**) reveals simultaneous positivity for S-100 protein and HLA-DR in most arborized cells both around intratumoral vessel (*) and within interstitium (arrows). Stellate morphology of HLA-DR/S-100 coexpressing cells is appreciated both along vascular sleeves (**h **and **j**) of tumoral microcirculation and as these tend to intimately commingle with adenoma cells (**i **and **k**). In order for reproducibility of specific labeling to be ensured, immunoreactions for both epitopes have been duplicated while interverting chromogens. Unique to the prolactin cell adenoma in Case 2 is the finding of adenomatous follicles (**l **and **m**) lined in part by HLA-DR immunoreactive FSCs. While rare follicles with nearly identical morphology also did stain for GFAP (**n**), actual coexpression could not be demonstrated. Immunohistochemical specimens depicted in **b **– **d**, and **m **– **n **were developed with horseradish peroxidase and 3,3'-diaminobenzidine; slides shown in **e **– **f**, and **l **are visualized using streptavidin-biotin-complex/alkaline phosphatase and new fuchsin-naphtol AS-BI as chromogen. In the double labeling studies (**g **through **k**), antigen names typeset in either red or black indicate fuchsin-naphtol and diaminobenzidine, respectively. Original magnification: **a**, **l **through **n **– ×200; **b **through **f **– ×100; **g **– ×400; **h **through **k **– ×1000 (oil immersion).

Our group has recently described a pituitary prolactinoma (index Case 1) with selective intratumoral infiltration by T lymphocytes, a phenomenon not documented previously [8-10]. This constellation has been interpreted as being consistent with a *forme fruste *of tumor immunosurveillance [11]. In an effort for that serendipitous finding to be addressed in a systematic context, a further fifty consecutive unselected adenomectomy specimens were screened for the presence of inflammatory infiltrates. Two additional occurrences – a sparsely granulated prolactin cell adenoma from a 31-year-old female (Case 2) and a gonadotroph cell adenoma of oncocytic type from a 67-year-old male (Case 3) – have been identified. None of the patients presented evidence of autoimmune endocrine disease nor was there clinically or radiologically detectable sinusitis. Both the morphology and immunophenotype of the intratumoral infiltrates closely paralleled those described in index Case 1 [8]: i.e., perivascular cuffs presided over by T lymphocytes with a predominance of CD4^+ ^cells that tended to commingle with S-100 protein immunoreactive process bearing cells (Fig. 1a–f). In addition, a fair number of the latter were scattered throughout the adenoma tissue. As originally described, neither ongoing necrosis nor fibrous replacement of the parenchyma was seen in these examples, either. Conspicuously, most – if not all – S-100^+ ^arborized cells also stained intensely for HLA-DR on immunohistochemical double labeling (Fig. 1g–k). Yet markers of follicular dendritic cells (FDCs) of lymphoid type (CD21) as well as Langerhans cells (CD1a) tested negative [12]. Furthermore, there was no staining for cytokeratins (MNF-116; CAM 5.2). While immunoreactivity for GFAP in arborized cells was largely absent as well, scattered adenomatous follicles lined by GFAP^+ ^FSCs in Case 2 did also reveal some staining for HLA-DR (Fig. 1l–n).

We read the present findings as ones suggesting selective involvement of the DC-like subset of FSCs in tumor immunosurveillance of pituitary adenomas, in particular as these fulfill the immunophenotypic criteria set forth by Allaerts et al [6,11]. Uncommon though this paradigm obviously is, it is felt to be offering the observational advantage of a rather restricted inflammatory reaction – one that may not be forthcoming in conventional lymphocytic hypophysitis which tends to involve a higher degree of vascular permeability as well as necrosis [13]. In the present setting, FSCs are perceived as ones – at least by immunophenotypic standards – capable of displaying tumor-related epitopes in the context of MHC class II antigens for native T lymphocytes. Henceforth, the defining cellular ingredients of "organized lymphatic tissue" in secondary lymphoid organs – to which tumoral antigens of pituitary adenomas are unlikely to gain access – may be granted a *de novo *local compartment at which to convene [11,12].

As to what extent FSCs of DC type actually are to be considered "professional" antigen presenting cells is, however, uncertain; especially with respect to their capacity to prevent apoptosis of selected clones of lymphocytes or cross-priming – functions routinely performed by FDCs. Both diagnostic experience with and the natural history of pituitary adenomas suggest that antitumoral immune defense eventually falls short of achieving proficient elimination of adenoma cells in most cases.

The findings reported above nevertheless are felt to indicate that (i) pituitary adenomas may not necessarily be ignored by immune recognition; (ii) immunosurveillance of pituitary adenomas involves an inflammatory compartment reminiscent of organized lymphoid tissue; and (iii) a subset of FSCs with DC phenotype are likely to perform a locum antigen presenting function.

The focal finding in Case 2 of stellate cells expressing HLA-DR, S-100 protein and GFAP in suggestive association along adenomatous follicles further raises the question whether FSCs of DC type are apt to either transdifferentiate into or else being recruited from their "classical/epithelial-like" or "classical/astrocyte-like" counterparts. Future characterization of the immunoprofile of spindle cell oncocytoma, a purported FSC neoplasm, may be expected to yield some insights into this topic [14].

Keeping in mind that a non-negligible part of pituitary adenomas are not ultimately amenable to cure by either conventional pharmacotherapy or surgery, exploring the prospect of immunomodulation may also be of interest in two respects. On the one hand, the physiologic potential of FSCs of DC type to negatively influence adenohypophyseal secretion invites to be taken advantage of by pharmacological induction to inhibit excess hormone secretion by adenomas [2,3]. On the other hand, a minimal formal framework of cell-mediated antitumoral defense obviously does emerge in a subset of adenomas. Converting such jejune inflammatory response into proficient antitumoral defense ought to be elaborated on in the larger context of tumor immunotherapy. Intriguingly enough, sporadic empirical reports on beneficial impact of T lymphocyte stimulation on the evolution of some pituitary adenomas are on record [15]. Further work dedicated to characterization of the inflammatory response in these tumors seems, therefore, warranted.

## Competing interests

The author(s) declare that they have no competing interests.

## Authors' contributions

The theoretical and technical framework of the study have been conceived by IV and AK. The clinical data have been compiled and critically interpreted by RS. The immunohistochemical studies have been supervised, and double labeling experiments personally carried out by AK. All three authors actively contributed to the composition of the manuscript, based on a draft by IV. The final version of the manuscript has been read and approved by all authors.
